# Effect of a Virtual Patient Navigation Program on Behavioral Health Admissions in the Emergency Department: A Randomized Clinical Trial

**DOI:** 10.1001/jamanetworkopen.2019.19954

**Published:** 2020-01-29

**Authors:** Jason Roberge, Andrew McWilliams, Jing Zhao, William E. Anderson, Timothy Hetherington, Christine Zazzaro, Elisabeth Hardin, Amy Barrett, Manuel Castro, Margaret E. Balfour, James Rachal, Constance Krull, Wayne Sparks

**Affiliations:** 1 Center for Outcomes Research and Evaluation, Atrium Health, Charlotte, North Carolina; 2 Behavioral Health Service Line, Atrium Health, Charlotte, North Carolina; 3 Department of Psychiatry, Atrium Health, Charlotte, North Carolina; 4 Department of Psychiatry, University of Arizona, Tucson

## Abstract

**Importance:**

The number of patients presenting to emergency departments (EDs) for psychiatric care continues to increase. Psychiatrists often make a conservative recommendation to admit patients because robust outpatient services for close follow-up are lacking.

**Objective:**

To assess whether the availability of a 45-day behavioral health–virtual patient navigation program decreases hospitalization among patients presenting to the ED with a behavioral health crisis or need.

**Design, Setting, and Participants:**

This randomized clinical trial enrolled 637 patients who presented to 6 EDs spanning urban and suburban locations within a large integrated health care system in North Carolina from June 12, 2017, through February 14, 2018; patients were followed up for up to 45 days. Eligible patients were aged 18 years or older, with a behavioral health crisis and a completed telepsychiatric ED consultation. The availability of the behavioral health–virtual patient navigation intervention was randomly allocated to specific days (Monday through Friday from 7 am to 7 pm) so that, in a 2-week block, there were 5 intervention days and 5 usual care days; 323 patients presented on days when the program was offered, and 314 presented on usual care days. Data analysis was performed from March 7 through June 13, 2018, using an intention-to-treat approach.

**Interventions:**

The behavioral health–virtual patient navigation program included video contact with a patient while in the ED and telephonic outreach 24 to 72 hours after discharge and then at least weekly for up to 45 days.

**Main Outcomes and Measures:**

The primary outcome was the conversion of an ED encounter to hospital admission. Secondary outcomes included 45-day follow-up encounters with a self-harm diagnosis and postdischarge acute care use.

**Results:**

Among 637 participants, 358 (56.2%) were men, and the mean (SD) age was 39.7 (16.6) years. The conversion rates were 55.1% (178 of 323) in the intervention group vs 63.1% (198 of 314) in the usual care group (odds ratio, 0.74; 95% CI, 0.54-1.02; *P* = .06). The percentage of patient encounters with follow-up encounters having a self-harm diagnosis was significantly lower in the intervention group compared with the usual care group (36.8% [119 of 323] vs 45.5% [143 of 314]; *P* = .03).

**Conclusions and Relevance:**

Although the primary result did not reach statistical significance, there is a strong signal of potential positive benefit in an area that lacks evidence, suggesting that there should be additional investment and inquiry into virtual behavioral health programs.

**Trial Registration:**

ClinicalTrials.gov identifier: NCT03204643

## Key Points

**Question:**

Does offering virtual patient navigation reduce admission rates for patients presenting to the emergency department with a behavioral health crisis?

**Findings:**

In this randomized clinical trial, there were fewer admissions on days when the navigation program was available (55.1%) vs on days with usual care (63.1%), although the difference was not statistically significant. Significantly fewer patients who used the navigation program had a follow-up encounter involving a self-harm diagnosis within 45 days compared with patients who received usual care (36.8% vs 45.5%).

**Meaning:**

Although the primary result did not reach statistical significance, there is a strong signal of potential positive benefit in an area that lacks evidence, suggesting that there should be additional investment and inquiry into this area.

## Teaser

This randomized clinical trial assesses whether the availability of a 45-day behavioral health–virtual patient navigation program decreases hospitalization among adult patients presenting to the emergency department with a behavioral health crisis or need.

## Introduction

Psychiatric care is frequently provided in emergency departments (EDs) for patients with mental health crises, resulting in long ED lengths of stay, hospital admissions, and high recidivism rates. In North Carolina, for example, 27% of patients discharged from the ED return in 30 days.^1^ Using the ED as a primary source of care affects both patient care quality and use of scarce health system resources.

Reduced access to integrated community-based mental health resources, in the face of pressures to move away from deinstitutionalization, has been associated with this growing crisis in mental health care.^2^ Owing to the lack of both coordinated resources and timely, appropriate follow-up, many patients with mental health crises are admitted unnecessarily to an inpatient psychiatric facility.^2^ Different strategies have been developed to help treat patients in mental health crises, including the use of community crisis centers to provide a buffer between outpatient facilities and EDs, the development of separate units within medical EDs for psychiatric patients,^3,4,5^ and the use of telebehavioral health consultations in primary care and EDs and freestanding dedicated psychiatric-only EDs.^6,7,8^

Because patients without at least 1 scheduled outpatient appointment after hospital discharge were twice as likely to be readmitted, others have worked to improve postdischarge follow-up.^9^ One study that evaluated periodic telephone check-ins after ED discharge demonstrated a 30% reduction in suicide attempts.^10^ A patient navigation program has also been suggested as a potential way to address gaps in community-based follow-up and fragmentation.^11^ In a pilot study, a peer navigator intervention was shown to change patients’ perspectives on the use of EDs as a source for primary care.^12^

Atrium Health covers 2.3 million patients across North Carolina, South Carolina, and Georgia and provides behavioral health services for 22 EDs. Atrium Health would be unable to place an in-person navigator intervention in every ED, making a telehealth or virtual model essential for feasibility and scale. The existing infrastructure offers telepsychiatric consultations in the EDs for patients in a behavioral health crisis; however, a patient follow-up program after discharge was not available.

The Hospital Improvement Innovation Network is a nationwide effort coordinated by the Centers for Medicare & Medicaid Services to reduce preventable hospital-acquired conditions and hospital use. As a participant in the Hospital Improvement Innovation Network, Atrium Health’s goal was to develop effective strategies to reduce unnecessary behavioral health hospitalizations and readmissions. Even if a patient can receive a psychiatric evaluation in the ED, it is challenging to provide effective treatment in such an environment because treatment often requires time, a therapeutic milieu, group interaction, and a calming environment. Most ED clinicians have 2 choices—discharge the patient back to his or her current social situation or admit the patient. For the reasons listed, little stabilization happens in the ED, so the default recommendation is often a hospital admission, leading to high admission rates. This study assesses the availability of a program that offers additional support. Some of the patients who would have defaulted to an admission can be discharged with the extra support. This program is an option for EDs and communities that do not have a dedicated crisis center or, as is the case in many communities with a center, when demand for crisis mental health care outweighs supply.

## Methods

### Objective

The primary aim of this randomized clinical trial, conducted from June 12, 2017, through February 14, 2018, was to determine whether the availability of a virtual outpatient program for ED-based telepsychiatric consultation decreased hospitalization among patients presenting with a behavioral health crisis. The intention was not to evaluate the efficacy of the program for those who participated. The potential for this program to reduce admissions is that the consulting psychiatrist would be more confident recommending discharge on days when he or she knows that the behavioral health–virtual patient navigation (BH-VPN) program is available for the patient (Figure). Patients who presented on intervention days (n = 323) received the same behavioral health clinician evaluation and telepsychiatry consultation as patients who presented on usual care days (n = 314). The study was approved by the Advarra Institutional Review Board, which granted a waiver of patient consent because they determined the research posed no more than minimal risks to participants and that the waiver would not adversely affect their rights and welfare. The full trial protocol is available in Supplement 1. This study followed the Consolidated Standards of Reporting Trials (CONSORT) reporting guideline.

**Figure.**
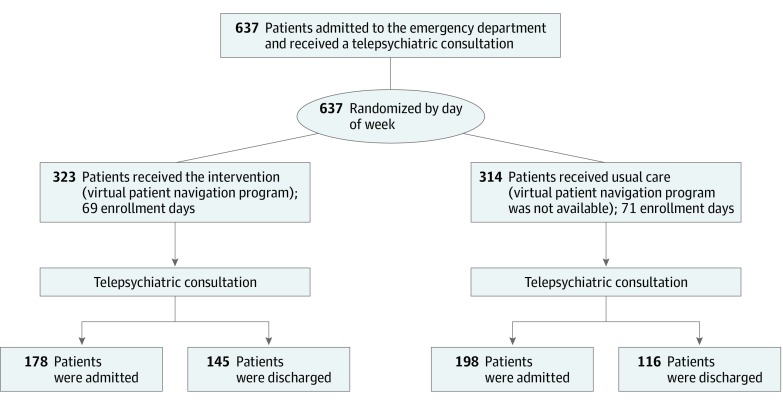
CONSORT Flow Diagram of Patients

### Study Setting

In this randomized clinical trial, the unit of randomization was the day on which the patient navigation intervention was available, creating an approximately equal number of days when the BH-VPN program was available (intervention group) and days when it was not available (control group). Six EDs spanning urban and suburban locations within Atrium Health participated in this study from June 12, 2017, through February 14, 2018. Patients were followed up for up to 45 days after ED discharge. Two EDs are freestanding, while the rest are physically connected to hospitals. At the onset of the study, 2 EDs participated, with the addition of 4 EDs after 2 months to match patient volume to navigation team capacity.

### Eligibility

Patients who met the following criteria were eligible: (1) presented to a participating ED and were in need of a psychiatric consultation for behavioral health crisis as deemed by the ED physician, (2) completed a telepsychiatric consultation as captured in the electronic medical record, (3) initiated telepsychiatric consultation Monday through Friday from 7 am to 7 pm (when BH-VPN is available for the intervention group), and (4) were aged 18 years or older at time of ED admission.

Patients with neurocognitive disabilities, such as dementia, were not eligible to receive services from the BH-VPN program. The intervention was designed for patients with normal cognition in large part because the navigators needed to be able to connect reliably via telephonic communication. The outpatient care needs of patients with cognitive disorders such as dementia differ from the care needs of patients with mental health disorders or substance use disorders. Many neurocognitive disorders are degenerative and progressive, with issues unlikely to be solved by having close follow-up with a virtual navigator. However, because there was not a reliable way to nondifferentially exclude patients with a neurocognitive disability from both study groups, they were included in the intention-to-treat analysis.

### Intervention

On intervention days, the BH-VPN program was made available for patient enrollment. The navigator introduced herself via video in the ED to patients who were deemed eligible for discharge to initiate a therapeutic rapport, enrolled the patient in the program, and explained the navigation services. The communication strategy was established in partnership with the patient, including best contact time, telephone numbers, inclusion and exclusion of supportive persons, and types of behavioral health follow-up indicated by patient self-report and psychiatry consultation recommendations. Barriers to treatment were also explored to ensure that referrals to appropriate community resources were provided. Referrals included, but were not limited to, counseling, psychiatry, primary care clinicians, transportation, medication resources, care management programs, food banks, and substance abuse treatment resources. Referrals were made in the context of the patient payer source, geography, and preferences of the patient. The navigators consisted of 2 licensed behavioral health clinicians (including E.H.) experienced in working with patients in crisis and community behavioral health resources.

The intervention was designed to ensure close and regular follow-up. Having such a program available could be reassuring to a telepsychiatric clinician who otherwise is unsure when or if the patient will follow up, as he or she tries to decide whether to admit or discharge the patient. Through a qualitative evaluation that will be published separately, we have had clinicians report that they are much more comfortable discharging a patient if it is known that a navigator is available to check in on the patient and make sure any appointments are timely.

Patients received a follow-up telephone call by a navigator within 24 to 72 hours from ED discharge, then at least weekly for up to 45 days. One or both navigators could follow up with a patient during his or her enrollment. A follow-up assessment was completed at each telephone contact, which included a suicide ideation safety screening (Columbia Suicide Severity Rating Scale Reassess),^13^ an appointment reminder, appointment barrier evaluation, medication obtainment follow-up, substance use disorder follow-up, supportive listening, psychoeducation, community resource follow-up, and additional crisis planning. If a patient was deemed to be actively suicidal, then the navigator recommended transfer to a mobile crisis unit, called 911 for a police officer to check on the patient (well-check), or called 911 to take the patient to the ED as per the current standard of care. Patients completed their participation 45 days from ED discharge or earlier if (1) their behavioral health crisis resolved, (2) an existing care management program unrelated to the study assumed responsibility, (3) they opted to discontinue the program, (4) they could not be reached after ED discharge (4 calls in 12 days), or (5) they declined participation prior to ED discharge.

### Usual Care

Usual care consisted of a licensed behavioral health clinician (nonphysician) completing an initial telephone assessment with all patients referred by the ED physician for telepsychiatric consultation. The behavioral health clinician obtained information on recent stressors, dangerous thoughts or behaviors, history of psychiatric treatment, social history, and collateral information. After this preliminary information gathering, patients were then seen by a psychiatrist using a video platform. In the virtual consultation, the psychiatrist interviewed each patient, completed a detailed mental status examination as well as a suicide risk assessment based on the information obtained in the Columbia Suicide Severity Rating Scale,^14^ and provided a diagnosis with treatment recommendations to the ED physician.

When inpatient psychiatric treatment was recommended but no bed was available for the patient at the time of consultation, the psychiatrist completed initial orders for psychiatric medication and monitoring, to begin immediately in the ED. For patients who did not require inpatient psychiatric treatment, the psychiatrist provided medication recommendations. In addition, the health care system’s guidelines recommended that the ED physician provide contact information to the local county or area mental health facility or substance treatment facility, and/or follow up with a primary care clinician.

### Outcomes

The primary outcome was conversion from ED to hospital admission. Admission data were obtained through the health care system’s existing medical record abstraction process, which populates a quality improvement registry. The registry enables centralized, multistate bed management for behavioral health inpatient units. The abstraction personnel were unaware of the study. Leveraging this registry allowed for the capture of data on admissions, both within and external to Atrium Health.

Secondary outcomes assessed acute care use consisting of inpatient, observation, and ED encounters to any Atrium Health facility. We examined single-site and multisite 30-day admission rates (both inpatient only and inpatient plus observation) and 45-day use rates (inpatient, observation, and ED). The 45-day rate of return to an ED with a telepsychiatric consultation was also examined. Death records and *International Statistical Classification of Diseases and Related Health Problems, Tenth Revision* billing codes for self-harm (R45.851, R45.850, T50.902A, X78.8XXA, T14.91, T42.4X2A, X83.8XXA, T43.502D, T43.592A, T43.202A, T65.92XA, T43.502A, X78.8XXD, X78.9XXA, T43.202D, T43.212A, and T43.222D) were collected.

The 45-day rate of follow-up encounters (inpatient or ambulatory) with a self-harm diagnosis was assessed. Consistent with the trial’s pragmatic design, and to allow for nondifferential outcomes assessment, all outcomes data were collected and available as part of routine care.^15^

### Safety Risks and Reporting

Independent behavioral health and research experts, including a statistician, served on a data and safety monitoring board, which followed a data and safety monitoring plan. Per this plan, the members reviewed the death rate and the 45-day rate of follow-up encounters with a self-harm diagnosis 3 months into the 6-month study, when the total sample size was 303, and recommended that the study continue as planned.

### Sample Size

This study was powered to detect a 15% absolute reduction in admissions with the usual care group, assumed to have a 50% admission rate (internal historical rate). To detect this reduction with 85% power, a total sample size of 414 (α = .05) was required, using the χ^2^ test for independence. To account for the possible correlation among patients seen in the ED on the same day, we inflated the sample size by 10%, assuming that the mean number of eligible patients per ED per day is 2, with a 0.1 intraclass correlation coefficient (design effect = 1 + [2 − 1] × intraclass correlation coefficient). Thus, the target sample size was 456. The power was calculated using PASS, version 15.^16^

### Randomization

In this randomized clinical trial, the unit of randomization was the scheduled day on which the patient navigation intervention was available. Patients accrued in both groups of the study, Monday through Friday from 7 am to 7 pm. Eligibility was based on the time stamp of the telepsychiatry consultation. All eligible patients in all participating EDs accrued into the group assigned for that day. Block randomization was used to allocate days to intervention or usual care using SAS Enterprise Guide, Version 7.1 for Windows on platform 9.4.1.^17^ During each period of 10 business days, there was a balance of 5 intervention days and 5 usual care days. The study statistician created the randomization list and provided the days on which the intervention was available to the behavioral health program coordinator. Telepsychiatrists were alerted by the coordinator that the program was available on intervention days. One intervention day fell on a holiday with the BH-VPN program not being available. There were 69 intervention days and 71 usual care days.

### Statistical Analysis

An intention-to-treat approach was used for all analyses. Patients meeting the inclusion criteria were evaluated as either having an admission or discharge from the ED. Distributions of baseline characteristics and safety outcomes were compared between the intervention and usual care groups, using the χ^2^ test for categorical variables and the *t* test for continuous variables. The primary outcome, conversion from ED encounter to hospital admission (yes or no), was analyzed using a generalized linear mixed model with a log link function. The models included a random effect for day nested within ED to adjust for correlation among patients seen on the same day in the same ED. Study group and presenting ED were considered fixed effects in the model. Results for group comparisons were presented with odds ratios and 95% CIs. This same approach was used to analyze 30-day and 45-day postdischarge health care use. The remaining secondary outcomes were compared between study groups using the χ^2^ test. All *P* values were from 2-sided tests, and the results were deemed statistically significant at *P* < .05; the data were analyzed using SAS Enterprise Guide, version 7.1 (SAS Institute Inc).

## Results

### Characterization of the Population

Demographic characteristics were similar between the intervention (n = 323) and usual care (n = 314) groups (Table 1). The overall population had a mean (SD) age of 39.7 (16.6) years, 358 (56.2%) were men, and 239 (37.5%) were self-pay. No patients were referred to a mobile crisis unit, and 911 was not needed for a well-check among participants in the intervention group because no patients were actively suicidal during any telephone call. Four patients receiving usual care died within 45 days of their telepsychiatric consultation, whereas no patients in the intervention group died. The deaths occurred outside of the hospital system, and the reasons are unknown.

**Table 1.** Demographic Characteristics and Safety Outcomes^a^

Characteristic	Participants, No. (%)	
	Intervention Day (n = 323)	Usual Care Day (n = 314)
Age, mean (SD), y	39.5 (16.8)	39.8 (16.3)
Male	183 (56.7)	175 (55.7)
Ethnicity		
Hispanic	24 (7.4)	19 (6.1)
Non-Hispanic	272 (84.2)	272 (86.6)
Unknown	27 (8.4)	23 (7.3)
Race		
African American	105 (32.5)	90 (28.7)
American Indian or Alaska Native	3 (0.9)	1 (0.3)
Asian	4 (1.2)	3 (1.0)
White	170 (52.6)	182 (58.0)
Multiracial	2 (0.6)	1 (0.3)
Other	34 (10.5)	28 (8.9)
Unknown	5 (1.6)	9 (2.9)
Health insurance		
Commercial or private	77 (23.8)	55 (17.5)
Medicaid	74 (22.9)	74 (23.6)
Medicare	49 (15.2)	57 (18.2)
Self-pay	116 (35.9)	123 (39.2)
Other or unknown	7 (2.2)	5 (1.6)
Self-harm within 30 d of telepsychiatric consultation^b^	113 (35.0)	134 (42.7)
Death within 30 d of telepsychiatric consultation	0	2 (0.6)
Self-harm within 45 d of telepsychiatric consultation^b^	119 (36.8)	143 (45.5)
Death within 45 d of telepsychiatric consultation	0	4 (1.3)

^a^ As a randomized trial, demographic characteristics were not compared with a statistical test. Safety measures were compared.

^b^ *P* < .05.

### Admissions and Health Care Use

The admission rate was 55.1% (178 of 323) on intervention days vs 63.1% (198 of 314) on usual care days (odds ratio, 0.74; 95% CI, 0.54-1.02; *P* = .06) (Table 2). In the generalized linear mixed model, the random-effect term of day nested within ED was not statistically significant; therefore, the final model included only the fixed effects of study group and ED.

**Table 2.** Primary and Secondary Results

Result	Participants, No. (%)		OR (95% CI)	*P* Value
	Intervention (n = 323)	Usual Care (n = 314)		
Conversion from ED encounter to hospital admission	178 (55.1)	198 (63.1)	0.74 (0.54-1.02)	.06
45-d all-cause, nonelective health care use at any hospital in Atrium Health				
Composite	112 (34.7)	89 (28.3)	1.35 (0.96-1.90)	.08
Inpatient	17 (5.3)	13 (4.1)	1.29 (0.61-2.69)	.50
Observation	19 (5.9)	19 (6.1)	0.97 (0.50-1.87)	.93
Emergency department	96 (29.7)	79 (25.2)	1.26 (0.89-1.78)	.20
45-d ED use with a telepsychiatric consultation at any ED in Atrium Health	53 (16.4)	49 (15.6)	1.07 (0.70-1.64)	.76
30-d inpatient, all-cause, nonelective health care use (same hospital in Atrium Health)	7 (2.2)	2 (0.6)	3.46 (0.71-16.76)	.18
30-d all-cause, nonelective health care use at any hospital in Atrium Health				
Composite	28 (8.7)	23 (7.3)	1.16 (0.65-2.08)	.62
Inpatient	16 (5.0)	10 (3.2)	1.58 (0.71-3.55)	.26
Observation	15 (4.6)	14 (4.5)	1.04 (0.50-2.20)	.91

Abbreviations: ED, emergency department; OR, odds ratio.

The 45-day all-cause, nonelective health care use (inpatient, observation, and ED) encounters comprised 34.7% (112 of 323) of the intervention group vs 28.3% (89 of 314) of the usual care group (odds ratio, 1.35; 95% CI, 0.96-1.90; *P* = .08) (Table 2). The 45-day postdischarge inpatient and ED admission rates were numerically but not statistically significantly higher in the intervention group (inpatient admission, 5.3% [17 of 323] vs 4.1% [13 of 314]; *P* = .50; ED admission, 29.7% [96 of 323] vs 25.2% [79 of 314]; *P* = .20), and the observation admission rates were similar (5.9% [19 of 323] vs 6.1% [19 of 314]; *P* = .93). The percentage of patients who had a 45-day postdischarge ED encounter with a telepsychiatric consultation was 16.4% (53 of 323) in the intervention group and 15.6% (49 of 314) in the usual care group (odds ratio, 1.07; 95% CI, 0.70-1.64; *P* = .76). Significantly fewer patients in the intervention group had a follow-up encounter involving a self-harm diagnosis within 45 days compared with patients in the usual care group (36.8% [119 of 323] vs 45.5% [143 of 314]; *P* = .03).

## Discussion

To our knowledge, this is the first randomized clinical trial to assess whether the availability of a virtual behavioral health program can reduce hospital admissions for patients presenting to the ED with a behavioral health crisis. Availability of the program resulted in an 8% absolute reduction in hospital admissions. Although this outcome was not statistically significant (*P* = .06) in the adjusted model, the odds ratio of 0.74, along with the reduction of self-harm encounters within 45 days, suggests the potential for positive patient outcomes and should guide future research directions. Our study was powered to show a 15% difference, so it is possible that a larger study is needed to achieve statistical significance for a smaller but still clinically significant effect size. For example, if an 8% reduction in hospital admissions were replicated across the health care system locally, or even nationally, it would help alleviate ED overcrowding and lead to increased downstream capacity in psychiatric inpatient facilities.

Given the potential patient risk associated with a mental health crisis, it is important for interventions targeted to this area to be evaluated rigorously not only for effectiveness but also for unintended consequences. For this reason, we included secondary outcomes of recidivism and harms across both groups with data and safety monitoring board oversight. Compared with the patients in the usual care group, the patients in the intervention group had 8.7% fewer follow-up encounters within 45 days for a self-harm diagnosis (*P* = .03). The intervention group also had a higher 45-day all-cause postdischarge health care use rate. Although this result was not statistically significant, the results were in the opposite direction than anticipated. One potential explanation is that patients in the intervention group were better connected to resources that prompted them to present to the hospital before they got to the point of self-harm.

### Limitations

The study has several limitations. First, the study was powered for a 15% absolute reduction in admissions, but we saw an 8% reduction. Given that an 8% reduction would still be clinically significant, future studies should be powered to detect this more conservative estimate of reduction. Second, using an intention-to-treat analysis allowed us to detect the overall effect on the at-risk population and limit biases; however, a consequence was significant crossover to usual care for patients with neurocognitive disability, who were not approached for the BH-VPN program. The results of the crossovers likely diluted the intervention effect. Third, treating clinicians and navigators were unable to be blinded to the intervention, but because the randomization scheme allowed for BH-VPN to be available only on specific days without the potential for individual manipulation of the offering, bias and contamination should be limited. Although the trial was at multiple sites (6 urban EDs), the setting was within 1 health care system, thus limiting generalizability. A deeper understanding of the effectiveness of intervention components and patient-reported outcomes is limited because we relied only on existing data that were readily extracted from the electronic health record. Additional context will be obtained through an ongoing qualitative study of health care professionals, clinicians, and patients who participated in the BH-VPN program.

## Conclusions

Although the primary outcome of admission reduction was not statistically significant, the results suggest the potential for meaningful clinical change in an area with high admission rates. Integrating virtual patient navigation into emergency mental health care delivery should be a focused priority area for future research.

## Notes

Supplement 1. **Trial Protocol**

Supplement 2. **Data Sharing Statement**
