# Supplementary material for: Effect of a Virtual Patient Navigation Program on Behavioral Health Admissions in the Emergency Department: A Randomized Clinical Trial
Source: JAMA Netw Open. 2020 Jan 29;3(1):e1919954. doi: 10.1001/jamanetworkopen.2019.19954 (PMC6991284; doi:10.1001/jamanetworkopen.2019.19954)

Protocol

TITLE: AN EVALUATION OF EMERGENCY DEPARTMENT TO ADMISSION CONVERSION AMONG PATIENTS WITH A TELEPSYCHIATRIC CONSULT WHO ARE FOLLOWED BY A BEHAVIORAL HEALTH – VIRTUAL PATIENT NAVIGATION TEAM

Coordinating Site:
Carolinas Medical Center
1000 Blythe Boulevard
Charlotte, NC 28203

Principal Investigator:
Wayne Sparks, M.D.
1000 Blythe Boulevard
Charlotte, NC 28203
Phone: (704) 355-5375
Email: Wayne.Sparks@carolinashealthcare.org

Co-Principal Investigator:
Jason Roberge, Ph.D., MPH
720 E. Morehead St.
Charlotte, NC 28202
Phone: (704) 355-0268
Email: Jason.Roberge@carolinashealthcare.org

Statistician:
Jing Zhao, PhD
1540 Garden Terrace, Charlotte, NC 28203
Telephone: (704) 355-1965
Email: Jing.Zhao@carolinashealthcare.org

The study will be conducted in compliance with the protocol, ICH-GCP and any applicable regulatory requirements.

Confidential

The information provided in this document is strictly confidential and is intended solely for the guidance of the clinical investigation. Reproduction or disclosure of this document - whether in part or in full - to parties not associated with the clinical investigation, or its use for any other purpose, without the prior written consent of the Sponsor-Investigator is not permitted.

Throughout this document, symbols indicating proprietary names (®, TM) are not displayed. Hence, the appearance of product names without these symbols does not imply that these names are not protected.

Protocol Approved By:

PRINCIPAL INVESTIGATOR:

____
Wayne Sparks, MD

____
Date

EXECUTIVE SPONSORS:

____
Print Name

____
Signature

____
Print Name

____
Signature

PROTOCOL SUMMARY	
**Study Title**	***HIIN – BH:*** *An evaluation of Emergency Department to admission conversion among patients with a telepsychiatric consult who are followed by a behavioral health - virtual patient navigation team*
**Study Design**	A randomized trial for quality improvement evaluation
**Study Objectives**	The primary objective is to evaluate the effect of a behavioral health virtual navigation team compared to usual care among patients with a telepsychiatric consult, on Emergency Department to inpatient conversion.<br><br>Secondary objectives of the evaluation include:<br>Compare the following between the intervention group and the usual care group:<br>o 45-day post discharge utilization (ED, inpatient, and observation encounters)<br>o CHS Quality, Comfort, and Care defined 30-day readmission rate. This is a readmission rate among patients with an inpatient readmission to the same CHS facility as the index encounter.<br>o The patient-centric defined 30-day readmission rate. This is a readmission rate among patients with an inpatient or observation readmission to any CHS facility.<br><br>Additional analyses will be to explore:<br><br>a. As a sub-analysis, qualitative outcomes will be collected and measured with patients, providers, and leaders. These outcomes will be collected and evaluated, per a sub-study protocol addendum.<br>b. Historical health records may be pulled to explore baseline rates of risk factors among prior behavioral health patients at CHS.
**Inclusion/ Exclusion Criteria**	Inclusion:<br>• 18 years of age or older<br>• ED visit<br>• Completed telepsychiatric consult at the ED visit<br>• Telepsychiatric consult occurs Monday through Friday during the Navigator's potential hours of operation
**Study Procedures**	Patients who present to participating CHS EDs with a telepsychiatric consult performed will be treated and followed per the proposed CHS behavioral health patient navigation pathway (BH-VPN) or usual care. As part of the current care process, Patients are identified by the ED physician in the ED as needing psychiatric evaluation and a referral is made to the telepsychiatric consult, the care of these patients will follow the allocated BH-VPN pathway or usual care. Regardless of participation in the BH-VPN pathway or usual care, the ED providers decide based on available information the ultimate disposition for patients --discharge from the ED or admit to the hospital.

	Patients who complete a telepsychiatric consult in the ED Monday through Friday during the hours of operation can be enrolled to either the control or intervention arm based on a randomization scheme that randomly allocates days that navigators are available. On days where the navigator is available, all patients who meet eligibility criteria will be considered exposed to the interventions; whereas, on days the navigator is not available all patients will be considered exposed to Usual care. Patients who are in the intervention arm will be offered navigation services for 45 days following ED discharge.<br>The BH-VPN pathway is defined by several key components:<br><br>• Introduction of the navigation process to the patient, while in the ED<br>• Evaluation of the patient and their needs.<br>• Follow-up evaluation within 72 hours by phone, and weekly contact with the BH-VPN until 45 days post ED discharge<br>• Confirm that a follow-up visit is scheduled and if the patient made it to the appointment.<br>• Placement into an appropriate case management program, if needed
**Statistical Analysis**	Analyses will include all patients identified as having completed telepsychiatric consult and meeting the additional inclusion and exclusion criteria. Comparisons of the intervention and usual care groups will be made using univariate analyses such as the t-test and chi-square test. The primary outcome, ED to inpatient conversion, will be compared between the two groups of patients using a generalized linear mixed model. Results will be presented with odds ratios and 95% confidence intervals.

LIST OF ABBREVIATIONS

AMI	Any Mental Illness
BH-VPN	Behavioral Health Virtual Patient Navigation
CHS	Carolinas Healthcare System
CORE	Center for Outcomes Research and Evaluation
C-SSRS	Columbia – Suicide Severity Rating Scale
DHHS	Department of Health and Human Services
EHR	Electronic Health Record
ED	Emergency Department
EDW	Enterprise Data Warehouse
FY	Fiscal Year
HIIN	Hospital Improvement Innovation Network
NC	North Carolina
OCTR	Office of Clinical and Translational Research
PHI	Private Health Information
QCC	Quality, Comfort, and Care
SOP	Standard Operating Procedures

TABLE OF CONTENTS

1 OBJECTIVES 8

1.1 Hypothesis 8
1.2 Primary Objective 8
1.3 Secondary Objectives 8

2 BACKGROUND 8
3 RATIONALE 10
4 SUBJECT AND SITE SELECTION 10

4.1 Accrual 10
4.2 Participating Sites .... Error! Bookmark not defined.
4.3 Inclusion\Exclusion Criteria 11
4.4 Evaluable Population 11

5 OVERALL DESIGN 11

5.1 Outcome Variables 11
5.2 Randomization and Allocation 13
5.3 Behavioral Health Virtual Patient Navigation (BH-VPN) 13
5.4 Follow-up 14
5.5 Patient Completion of Participation 14
5.6 Continuation of the Intervention 14

6 DATA COLLECTION AND REPORTING 14

6.1 Sample size analysis and statistical analysis 15
6.2 Statistical analysis 15
6.3 Data Collection Dates 15

7 PROJECT TIMELINE 17
8 INTERVENTION PLAN 16

8.1 Navigator Assessments 17
8.2 Follow-up Assessments 18

9 STUDY GOVERNANCE 18

9.1 Protocol and Pathway Training 17

10 SAFETY RISKS AND REPORTING 19

10.1 Data and Safety Monitoring Board (DSMB) 18
10.2 Data Quality Assurance 19
10.3 Safety Reporting to the IRB 19
10.4 Safety Monitoring by the Sponsor 19

11 RESEARCH COMPLETION **20**
12 ETHICAL AND LEGAL ISSUES **21**

12.1 Ethical and Legal Conduct of the Study **21**
12.2 Confidentiality **21**
12.3 Disclosure of Data **21**

12 RETENTION OF RECORDS **21**
13 PUBLICATION POLICY **22**
REFERENCES **22**
APPENDICES **23**

OBJECTIVES

1.1. Hypothesis

If behavioral health navigation is offered, there will be a lower ED to hospital admission conversion rate of patients who have had a telepsychiatric consultation in the ED, than if only usual care is available to patients.

1.2. Primary Objective

The primary objective is to evaluate whether the conversion from ED encounter to admission is changed when the virtual patient navigation program is available as compared to usual care in patients who have had a telepsychiatric consultation in the ED.

1.3. Secondary Objectives

The secondary objectives are to examine the behavioral health – virtual patient navigation team's effect on additional patient outcomes, such as the

i. CHS Quality, Comfort, and Care 30-day readmission rate. This is a readmission rate among patients with an inpatient readmission to the same CHS facility as the index encounter. This measure applies to patients that have a hospital admission subsequent the ED encounter.
ii. The patient-centric 30-day readmission rate. This is a readmission rate among patients with an inpatient or observation readmission to any CHS facility. This measure applies to patients that have a hospital admission subsequent the ED encounter.
iii. 45-day post ED discharge utilization (ED, inpatient, observation encounters)

2. BACKGROUND

Hospital admissions are common amongst those with mental illness. Significant morbidity exists for patients who are being admitted to a psychiatric hospital from the Emergency Department (ED). Additionally, admission to a hospital setting may have adverse effects on patients psychologically, degrade relationships with therapists, and disrupt continuity of care. Based on verbal reports from psychiatrists providing virtual consults at Carolinas HealthCare System (CHS), providers often decide to admit to a psychiatric hospital because of limited availability of outpatient behavioral health resources.

The number of patients seeking psychiatric services continues to grow at the national, state, and system level. In 2015, there were an estimated 43.4 million adults aged 18 or older in the United States with AMI (Any Mental Illness) within the past year. This number represented 17.9% of all U.S. adults [1]. In North Carolina, every 2.5 minutes a person in behavioral health crisis visits an ED [2]. For Carolinas Healthcare System's Metro EDs, Behavioral Health visits increased from 8,449 in 2011 to 14,293 in 2016 (internal report). In North Carolina's FY12, 19,020 persons seeking help in an ED for a primary mental health, developmental disability, or suicide attempt issue accounted for 26,009 visits, a repeat visit rate of 27%. Thirteen percent of those re-admissions occurred within 30 days. (DHHS data analysis of NC Medicaid Claims) [3]. Based on a Premier Quality Advisor Report that includes readmissions for 26 Carolinas

Healthcare System hospitals, patients who have a primary behavioral health diagnosis demonstrated a 7% readmission rate in 2015.

As the number of behavioral health patients presenting to the acute care EDs for psychiatric care continues to increase, we find there is a significant need to find interventions that will avoid unnecessary admissions or readmissions of these patients. Through verbal reports, ED psychiatrists at CHS must make the decision to admit a patient because there is no one to facilitate an outpatient appointment, ensure medications are filled, or provide follow-up so the patient can be discharged. Hospital readmission within 30 days of discharge usually represents a negative clinical outcome for patients with mental disorders and may be due to factors such as poor access to adequate community-based aftercare and challenges in psychiatric medicine and self-care[4]. Some of these challenges could be addressed by patient navigation, which has been shown to be effective. The largest suicide intervention trial in the U.S has shown a 30 percent reduction in suicide attempts over a 1 year follow-up as compared to standard care in the ED. This intervention utilized safety planning periodic check-ins via a phone call[5].

Carolinas Healthcare System's began utilizing telepsychiatry in 1997. The success of the virtual model led to the development of the robust program that exists today. The CHS virtual model currently offers 21 Metro EDs 24-7 access to licensed clinicians and psychiatrists who are able to provide psychiatric evaluations and dispositions. A 2007 study showed psychiatric consultation and short-term follow up provided by telepsychiatry can produce clinical outcomes that are equivalent to those achievable when patients are seen face to face[6].

The Hospital Improvement Innovation Network (HIIN) is a nationwide effort to reduce preventable hospital acquired conditions and hospital readmissions. Part of the HIIN effort surrounding behavioral health patients, includes identifying packages or key components most effective at preventing psychiatric admission and exploring ways to efficiently apply effective strategies. As a complement to the telepsychiatry program, a partnership has been formed with HIIN to develop a patient navigator program to provide short-term follow up to patients who are evaluated by telepsychiatry and potentially eligible for discharge home. The patient navigator will connect with the patient virtually prior to discharge from the ED and assist the patient in obtaining services and overcoming any barriers for 45 days post ED discharge.

By providing this wrap-around service to complement Carolinas HealthCare System's telepsychiatry program, we expect to decrease the number of patients admitted for inpatient psychiatric treatment and increase the number of discharges from the ED by providing another layer of service to our providers and patients. Additionally, we aim to decrease hospital inpatient admissions and increase after-care compliance with medications and appointments through short-term follow up. In one study regarding inpatient psychiatric care, it was found that patients who did not comply with at least one outpatient appointment after discharge were two times more likely to be readmitted than those who kept at least one appointment after discharge[7].

3. RATIONALE

To enhance the care of patients with a telepsychiatric consult, Carolinas HealthCare System (CHS) has designed a Behavioral Health Virtual Patient Navigation pathway (BH-VPN). The BH-VPN will monitor patients and assist with navigation through a weekly phone call after their index ED encounter for up to 45 days post ED discharge. The BH-VPN aims to improve patient outcomes through standardized approaches that leverage analytics and technology, while bridging care coordination. When a patient is presents to a participating CHS ED sites, those with a telepsychiatric consult completed on a randomized intervention day, during the hours of operation, are provided the option of participating in the BH-VPN. A patient can choose not to receive care by the navigator post discharge.

The BH-VPN pathway includes the following key components: introduction to the patient follow-up process prior to ED discharge, follow-up evaluation within 72 hours by phone and weekly contact, confirmation that a follow-up visit is scheduled, where applicable confirm in-network follow-up appointments are completed with a CHS provider, and placement into an appropriate case management program, if needed. There may be cases where patients are eligible to enroll in full case management programs based on their insurance and certain criteria. For example, if a patient has Medicaid through Cardinal Innovations, a managed care organization providing local behavioral health care, and meets certain criteria, and would no longer require navigation services. A patient’s contact with the BH-VPN ends after 45 days following ED discharge, or if there is a failure to contact, becomes enrolled with a case-management program, declines services, or the patient dies. The navigator will ensure the patient is enrolled in a case management program through contact with staff before ending contact with the patient.

This research project is a pragmatic, randomized quality improvement evaluation which seeks to evaluate the effects of standardizing the use of a BH-VPN among patients with a telepsychiatric consult. The outcomes evaluation of this quality improvement intervention has been designed to integrate into the routine care and minimize frontline staff burden by deploying an evaluation in a real-world setting.

4. SUBJECT AND SITE SELECTION

4.1. Accrual

As part of current care, patients are identified by an ED physician in the ED as needing psychiatric evaluation, and a referral is made to the telepsychiatric provider for a virtual consult. Patients are eligible to be included in analysis for the project if they meet the inclusion\exclusion criteria and the telepsychiatric consultation occurs during potential BH-VPN hours. We will accrue patient data for evaluation during a period starting in June 2017. Based on data from 2016, we estimate 500 patients will complete a telepsychiatric consult in a 6-month period during potential BH-VPN hours of operation. Patients will not be accrued on days where the navigators could not potentially be available, such as nights, weekends, and holidays.

Once a patient is in the intervention arm, the patient remains in the intervention arm until 45 days, or if there is a failure to contact (4 phone calls in 12 days), if the patient becomes enrolled with a case

management program, declines services, or if the patient dies. After 45 days, a patient is eligible to be re-enrolled in either arm of the study.

A patient remains in the control arm for 45 days unless the patient returns to the ED on a day where patients are being randomized to the intervention arm.

4.2. Inclusion\Exclusion Criteria

4.2.1. Inclusion Criteria

Eligible patients must meet each of the following criteria:

- Present to an ED at participating sites
- Completed a telepsychiatric consult as captured in the electronic medical record
- Telepsychiatric consult completed Monday through Friday during the Navigator's potential hours of operation
- $\geq$ 18 years of age at time of ED admission

4.2.2. Exclusion Criteria

No exclusion criteria.

4.3. Evaluable Population

Patients included in the evaluable population for this project, will have their data inform the final outcomes assessment. All patients who meet the inclusion criteria and have a completed telepsychiatric consult will be assessed. On days of being randomized to intervention, all eligible patients will automatically receive part A of intervention (navigator being available at the ED). Patients admitted directly from the ED or have neurocognitive disabilities from the intervention arm will not receive part B of services from the BH-VPN. All patients in the both arms will be assessed for the primary outcome (intent to treat). Patients will have one of the following ED discharge dispositions: (admission to the hospital for medical reasons, admission to a behavioral health facility by voluntary or involuntary commitment, discharge from the ED, or death).

5. OVERALL DESIGN

5.1. Outcome Variables

5.1.1. Primary Outcome Variable

The primary outcome variable is ED to inpatient or observation conversion. This is a patient that presents to a CHS ED site and then are admitted for any reason to a CHS facility as an inpatient or observation patient A patient's disposition is tracked to the encounter's outcome (admitted within CHS, Admitted outside CHS, no admission). The tracking of a patient's disposition is done by a team outside the behavioral health team involved in this study.

5.1.2. Secondary Outcome Variable(s)

Secondary outcome variables of the evaluation include:

Examining the BH-VPN's effect on additional patient outcomes as compared to usual care, such as

a. CHS QCC defined 30-day readmission rate. This is a readmission rate among patients with an inpatient readmission to the same CHS facility as the index encounter. This measure applies to patients that have a hospital admission subsequent the ED encounter.
b. The patient-centric defined 30-day readmission rate. This is a readmission rate among patients with an inpatient or observation readmission to any CHS facility. This measure applies to patients that have a hospital admission subsequent the ED encounter.
c. 45-day post ED discharge utilization. Utilization is defined as an inpatient, observation, or ED encounter at a CHS facility.

5.1.3. Additional assessments

As a sub-analysis, qualitative outcomes will be collected and measured with patients, providers, and leaders. These outcomes will be collected and evaluated, per the sub-study protocol addendum. Patient details will be identified and pulled from the EMR for recruitment and data collection. Historical health records may be pulled to explore baseline rates of risk factors among prior behavioral health patients at CHS.

5.1.4. Adverse Events

Safety/Serious Adverse Events that will be specifically monitored/evaluated include self-harm and death. Self-harm is tracked by diagnosis codes of self-harm and suicide ideation within 30 days of the telepsychiatric consult. Death rates can be compared between the usual care and intervention populations once a month using data in the Enterprise Data Warehouse. Death is noted based on notification to CHS by a friend or family member or from identification of death from the social security death index which occurs through monthly updates.

5.2. Randomization and Allocation

Randomization for the purposes of this project, will be based on days the Navigators are available to treat patients who've completed their telepsychiatric consult per the BH-VPN or usual care. Patients may be in the ED for multiple days. Their enrollment status depends on the day and time of their initial telepsychiatric consultation. Navigation will only be available for those patients whose consult occurs during intervention day/times. As part of the research design and rollout of this project, randomization will occur at the day level with the days being identified as intervention or usual care days for the duration of the study. We expect an average of 3.7 patients seen daily for both hospitals combined. Patients will accrue in both arms of the study Monday through Friday during the hours of operation. Patients will not be accrued on days where the navigators are not available such as weekends, nights, and holidays.

5.3. Behavioral Health Virtual Patient Navigation (BH-VPN)

The BH-VPN outlines the flow of patients from initial contact to their follow-up calls (see Appendix 1). In the existing ED workflow patients are identified by an ED physician in the ED as in a behavioral health crisis and needing psychiatric evaluation. A referral is made to the telepsychiatric provider for a virtual consult performed on a tablet in the ED. The patient is placed either in usual care (the control) or BH-VPN (the intervention arm of the study). The placement is dependent upon the day and hours of the week, as to whether navigation is available. After virtual consultation is performed via tablet, the telepsychiatrist will recommend to the ED provider that the patient either be admitted or discharged from the ED, ultimately that decision lies with ED provider. If the patient is in the intervention arm does not have neurocognitive disabilities such as Alzheimer's and discharge is recommended, the patient will be approached by the virtual navigator. The patient has the option not to be followed by the navigator. The navigator will complete the initial virtual behavioral health assessment. The initial health assessment contains information such as preferred communication method, primary care provider, psychiatry provider, collateral resources, recent ED and hospital utilization, substance use disorder, medication barriers, appointment barriers, community resources, and crisis planning.

The follow-up assessments, which will occur during navigation phone calls every at least weekly includes information such as a suicide ideation safety screening, an appointment reminder, appointment barrier evaluations, medication obtainment follow-up, substance use disorder follow-up, supportive listening, psychoeducation, community resource follow-up, and additional crisis planning.

The Columbia-Suicide Severity Rating Scale (C-SSRS) helps identify whether someone is at risk for suicide, and the severity and immediacy of the risk. It also identifies level of support the person needs. The screening tool is administered during each phone contact to measure suicide ideation. If a patient is deemed actively suicidal then the navigator will recommend transfer to mobile crisis, calling 911 for a well-check, or 911 to go to the ED as per current standard of care.

5.4. Follow-up

Patients being treated on the BH-VPN will receive a follow-up phone call within 24 to 72 hours from discharge, and then weekly for up to 45 days. A follow-up assessment is completed upon each phone call.

5.5. Patient Completion of Participation

Patients who are treated per the protocol on the control or intervention arm will complete their participation after 45 days or if one of the following occurs:

- Unable to connect: calls attempted over 12 days (intervention arm only)
- Death
- Enrolled in another Care Management program (intervention arm only)
- Declined services

- 45 days post-discharge completed

A patient’s utilization may be tracked post 45 days after completion.

5.6. Continuation of the Intervention

Participating ED sites may continue treating patients with the BH-VPN after the study period has ended.

6. DATA COLLECTION AND REPORTING

Patient demographics, comorbid conditions, and utilization will be obtained from the electronic medical record and billing systems. Data from the initial assessment and follow-up contacts will be stored in HealtheCare, a care management platform in Cerner. Data will be retrieved by an application specialist on the research team. Data may be retrieved retrospective to a patient’s completion of the intervention or usual care arm of the study. A monthly executive summary will be produced, showing patient volume, percent patients with suicidal ideation, and utilization.

6.1. Sample size analysis and statistical analysis

We are conducting the study for about 6 months leading to approximately 6 months*4 weeks*5 days=120 days with 60 days allocated to intervention and 60 allocated to treatment as usual. This study is designed to detect a 15% absolute reduction in the inpatient admission within 5 days of index ED presentation with the usual care group assumed to have a 50% inpatient admission rate (internal report). We will have 85% power to detect this reduction with a total sample size of 414 (α=0.05) using a Chi-square test for independence. We will not need to adjust for attrition as the primary outcome of inpatient admission <5 days is obtainable from electronic health records for all individuals. To account for the possible correlation among patients seen in the ED on the same day, we have inflated the sample size by 10% assuming the average number of patients per ED per day is 2 and the intra-class correlation coefficient=0.1 (Design effect=1+(2-1)ICC). Therefore, our target sample size is N=456.

6.2. Statistical analysis

All analyses will follow intention to treat such that patients will be analyzed based on the allocated intervention on the day of their ED visit (BH-VPN or treatment as usual). We will compare the two groups on age, sex, suicide ideation, and neurocognitive disorders to assess for balance, which is inferred with randomization.

The primary outcome, inpatient admission, will be compared between the two groups of patients using a chi-square test. We will explore differences by site by conducting stratified analysis by hospital. We will attempt to adjust for the correlation among patients seen on the same day using a generalized linear mixed model with a log link and random effect for day, nested within hospital. If convergence problems arise due to small sample sizes, we will collapse by week rather than day. We will also

control for hospital as a fixed effect in the model. Results for group comparisons will be presented with odds ratios and 95% confidence intervals.

Secondary outcomes are 45-day post discharge utilization (yes/no), QCC defined 30-day readmission (yes/no), and patient centric 30-day readmission (yes/no). Each of the secondary outcomes are dichotomous and will be compared using the same approach as the primary outcome. All tests will be two sided and the data will be analyzed using SAS Enterprise Guide 6.1 (SAS Institute Inc., Cary, NC, USA). For all outcomes, we assume if there are no visits in the CHS electronic medical record that the value for having a visit is null.

Interim Analysis

All interim analysis results will be presented to the Data and Safety Monitoring Board who will make a recommendation on continuing the trial as planned or modified. The following analyses will be conducted by the study statistician.

1) Interim safety analysis with 50% information: We propose to conduct interim analysis on the safety outcomes of (1) acute care visits (ED, observation, inpatient) within 30 days of index ED visit for the following diagnoses: suicidal attempt, suicidal ideation, and self-harm, and (2) death. For the acute care visits, we propose these analyses once 50% of patients have been accrued in the trial and outcomes are available via electronic data pull which we believe will coincide when 75% of the total sample size is accrued. For the acute care visits for self-harm, we will use an alpha spending function with symmetric boundaries of Pocock and an overall conservative α=0.10 due to the severity of the safety outcome. The values of the boundaries are ±1.87 and ±1.88 at 50% and 100%, respectively.

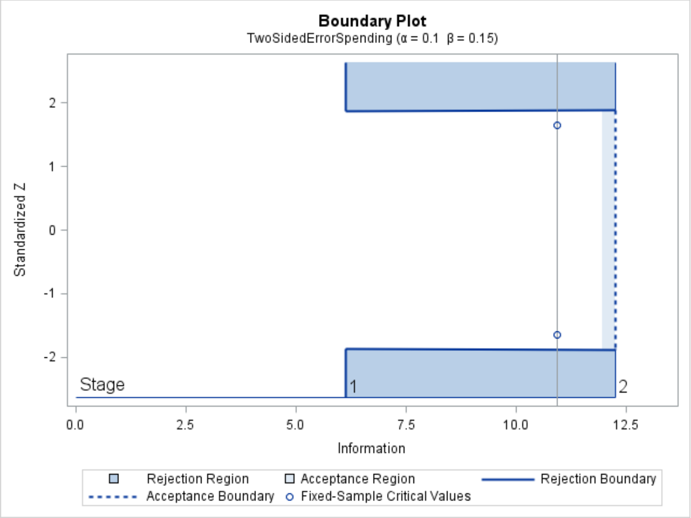

2) For the death analysis, the boundaries presented are only meant as *guidance*. Safety outcomes as severe as death do not have to meet the statistical boundaries for the DSMB to recommend stopping the trial for safety. We propose boundaries *within a single arm* based on a continuous monitoring algorithm which adjusts for repeated looks at the data based on a Pocock boundary (Ivanova, Qaqish, Schell, 2005). This method is based on the binomial distribution given the sample size and expected rate of death within 45 days. The boundaries below are provided for a design that yields the probability of crossing the boundary at most 10% when the rate of deaths is equal to 1%. These boundaries could be used at any interim look at the data where the information is available for the number accrued below.

Number accrue in trial and death information available	Boundary
1-3	1 deaths
4-26	2 deaths
27-66	3 deaths
67-116	4 deaths
117-171	5 deaths
172-228	6 deaths

6.3. Data Collection Dates

The implementation of the BH-VPN will begin in June 2017 among patients with a completed telepsychiatric consult.

7. PROJECT TIMELINE

**Project Timeline**	**2017**								**2018**				
	**May**	**June**	**July**	**Aug**	**Sept**	**Oct**	**Nov**	**Dec**	**Jan**	**Feb**	**Mar**	**Apr**	**May**
**Initial IRB Submission**	■	■											
**Site/Teammate Training (SIV)**	■	■											
**Prepare Methods manuscript**	■	■											
**Registration with Clinical Trials.gov**	■	■											
**First Patient on Study**		■											
**Interim Safety Analysis**								■					
**Last Patient on Study**								■					
**Study Duration**		■	■	■	■	■	■	■	■	■			
**Final Data Analysis**											■	■	■
**Internal white paper**													■
**Manuscript Prep and Submission**													■
**Conference Presentation/Posters**													■

8. INTERVENTION PLAN

8.1. Navigator Assessments

The initial assessment within 72 hours will capture the following components:

- Communication method
- Insurance
- Primary care provider
- Outpatient Psychiatry provider
- Collateral resource
- Recent visit summary
- Substance abuse
- Medication barriers
- Appointment Barriers
- Transportation needs
- Access to medications
- Community Resources
- Columbia-Suicide Rating Scale to assess for suicide ideation

8.2. Follow-up Assessments

The weekly follow-up contact assessments are comprised of the following components:

- Columbia-Suicide Rating Scale to assess for suicide ideation
- Appointment reminder\follow-up
- Medication access barriers
- Substance use disorder follow-up
- Supportive listening
- Psychoeducation
- Utilization of community resources
- Crisis planning and adherence to the plan by the patient

9. STUDY GOVERNANCE

This quality improvement trial will be conducted at Carolinas HealthCare System. It will be run jointly by the Center for Outcomes Research and Evaluation (CORE) and the Behavioral Health Department. Wayne Sparks, MD, (ED Psychiatry) will serve as the Principal Investigator with oversight from the Executive Committee (EC). Jason Roberge, PhD, MPH will serve as co-principal investigator on behalf of CORE. The EC will consist of leaders across the System involved in the trial, quality improvement, and implementation (Table 1). The EC will have the overall responsibility of trial oversight and direction. The EC will support dissemination of project findings and next steps. The EC will receive progress reports and will meet periodically for status updates from the team and to set direction. When appropriate, ad hoc committee meetings will be scheduled to discuss pressing concerns.

Table 1. Executive Committee	
Wayne Sparks	Behavioral Health
Manuel Castro	Behavioral Health
Scott Furney	CHS Executive Leadership/Internal Medicine
Mary N. Hall	CHS Executive Leadership
James Hunter	CHS Executive Leadership
Scott Rissmiller	CHS Executive Leadership

9.1. Protocol and Pathway Training

Background, protocol and process steps will be presented to psychiatrists and nurse practitioners who will be providing virtual psychiatric consultations. The role of virtual patient navigator and randomization scheme will be presented so expectations are clear that this is not available every day/time or weekends. This will be presented at the Department of Psychiatry meeting, which includes Emergency and Telepsychiatry Departments. Attendees may also include providers, who at some point may see patients who have been through this pathway, but will not be directly involved in providing evaluations. Education will be provided to the emergency department staff involved in this project, so that they are aware of the process and the role of the virtual patient navigator.

10. SAFETY RISKS AND REPORTING

The data collection and intervention for this project presents no more than minimal risk to patients. However, patients who present to the ED with a behavioral health crisis are considered a high-risk population. The implementation of the BH-VPN and its components complement ongoing patient care through virtual patient navigation within CHS. The addition of an evaluation design that aligns with existing patient care where there are limited resources thus confers minimal additional risk to patients. Resource constraints do not allow for the virtual patient navigation to be offered on all days allowing for a natural experimental design.

Implementation of new innovative techniques in the usual care process potentially introduces an increased possibility for care elements that are less effective, as effective, or more effective in providing quality of care. To address additional risk, or increased time without direct visual and in-person medical supervision we will collect the following potential adverse events: deaths, and reported events of suicidal ideation. These events will be weighed against average expected rates in this population by independent providers and experts in behavioral health and research, who will serve on a data and safety monitoring board.

Other potential risks of participation in this project include, the risk of health information disclosure. There is always the risk of disclosure of a patient’s private health information (PHI) or medical information. However, the processes identified in this protocol to enable the execution of this project, do not increase inherent risk of disclosure. Carolinas HealthCare System utilizes several hard and soft safety controls in the protection of patient information and medical records. Security controls include, but are not limited to, multiple system firewalls, access restrictions to patient records and information, locked offices and buildings housing research and patient data, and multiple layers of username and password protected computer and system access. The project team will ensure that appropriate handling of patient PHI follows standard CHS procedure. In the event of PHI disclosure, the appropriate internal departments will be informed and processed per legislation and privacy regulations.

10.1. Data and Safety Monitoring Board (DSMB)

Per the NIH, A DSMB is an independent advisory body of experts appointed to assess, at regular intervals, the progress of a trial, review accumulating data, evaluate safety event reports, and determine critical efficacy endpoints in a manner that contributes to the safety of subjects and the continued validity and scientific merit of the trial. Due to the project’s high-risk population, this protocol will be monitored according to the protocol-specific data and safety monitoring plan, and will abide by standard operating procedures set forth by both the Carolinas Healthcare System Office of Clinical and Translational Research and CORE. It is the responsibility of the Principal Investigator to monitor the safety data for this study. The Principal Investigator, Statistician, and other team members will meet as needed to review enrollment and retention, safety data for all subjects, study progress, and validity/integrity of the data. Documentation of these meetings will be kept with study records. The Principal Investigator will submit data to the project-specific Data and Safety Monitoring Board according to the overarching Data and Safety Monitoring Plan.

10.2. Data Quality Assurance

This study will be organized, performed, and reported in compliance with the study protocol, standard operating procedures (SOPs) of the CORE and CHS OCTR, and other applicable regulations and guidelines (e.g. GCP).

10.3. Safety Reporting to the IRB

All events occurring during the conduct of a protocol and meeting the definition of a reportable safety event per the Chesapeake IRB guidelines, will be reported to the IRB within 10 working days of the Investigator learning of the event, per their requirements.

Major protocol deviations that result in a threat to subject safety or the integrity of the study will be reported to the IRB per their requirements.

10.4. Safety Monitoring by the Sponsor

The conduct of this project will abide by standard operating procedures set forth by both CHS OCTR and CORE. It is the responsibility of the Principal Investigator to monitor the safety data for this study. The Principal Investigator, Statistician, and other team members will meet as needed to review enrollment and retention, safety data for all subjects, study progress, and validity/integrity of the data. Documentation of these meetings will be kept with study records.

11. RESEARCH COMPLETION

The Principal Investigator has the right to close the project at any site any time.

For any of the above closures, the following applies:

- Closures should occur only after consultation between involved parties.
- All affected institutions must be informed as applicable, according to local law.
- In case of a partial study or site closure, patients still participating in the COPD clinical pathway, or those who are considered in follow-up, must be taken care of in an ethical manner.

The study will be considered complete when one or more of the following conditions is met:

- The enrollment period has ended, and the data collection period is complete.
- All subjects have dropped out or discontinued from the study after the enrollment period is completed, but prior to data collection cutoff as described in section 8.3.
- The IRB, DSMB, or Principal Investigator discontinues the study.
- The Principal Investigator defines an administrative or clinical cut-off date.
- The DSMB deems the study inefficacious or unsafe.

Upon study completion, a final report will be presented to the Executive Committee and all key stakeholders. The final report will detail all findings including primary, secondary and exploratory

outcomes. The team will also prepare a manuscript for publication focused on outcomes and feasibility of implementation of the transition clinic.

12. ETHICAL AND LEGAL ISSUES

12.1. Ethical and Legal Conduct of the Study

The procedures set out in this protocol, pertaining to the conduct, evaluation, and documentation of this study, are designed to ensure that the Investigators abide by Good Clinical Practice (GCP) guidelines and under the guiding principles detailed in the Declaration of Helsinki. The study will also be carried out in keeping with the applicable local laws and regulation(s).

Documented approval from appropriate agencies (e.g. IRB) will be obtained before the start of the study, per GCP, local laws, regulations, and organizations.

Strict adherence to all specifications laid down in this protocol is required for all aspects of study conduct; the Investigators may not modify or alter the procedures described in this protocol.

Modifications to the study protocol will not be implemented without consulting the Principal Investigator and the IRB, as applicable. The Principal Investigator must assure that all study personnel, including co-investigators and other study staff members, adhere to the study protocol and all applicable regulations and guidelines regarding research both during and after study completion.

The Principal Investigator will be responsible for assuring that all the required data will be collected and properly documented.

12.2. Confidentiality

All records identifying the subject will be kept confidential and, to the extent permitted by the applicable laws and/or regulations, will not be made publicly available.

12.3. Disclosure of Data

The Principal Investigator, his associates and co-workers, and the appropriate regulatory agencies may use the information and data included in this protocol as necessary for the conduct of the study. Information contained in this study, and data and results from the study are confidential and may not be disclosed without the written permission of the Principal Investigator.

13. RETENTION OF RECORDS

Essential documentation including all IRB correspondence, will be retained for at least 2 years after the investigation is completed. Documentation will be readily available upon request.

14. PUBLICATION POLICY

The Principal Investigator or designee must send any draft manuscript, abstract, or conference presentation to members of the project Executive Committee for feedback and transparency, prior to submission of the final version. The Principal Investigator will be responsible for all relevant aspects regarding data reporting and publication.

The Principal Investigator or designee will ensure that the information and results regarding the study will be made publicly available on the internet at www.clinicaltrials.gov.

REFERENCES

1. Any Mental Illness (AMI) Among U.S. Adults. at https://www.nimh.nih.gov/health/statistics/prevalence/any-mental-illness-ami-among-us-adults.shtml.)
2. Relations NP. NC Behavioral Health By The Numbers. 2016.
3. Vigod SN, Kurdyak PA, Dennis C-L, et al. Transitional interventions to reduce early psychiatric readmissions in adults: systematic review. The British Journal of Psychiatry 2013;202:187-94.
4. North Carolina’s Mental Health Crisis [Fact Sheet]. 2014. at http://crisissolutionsnc.org/wp-content/uploads/2014/01/CSIfactsheet12-27-13.pdf.)
5. Miller IW, Camargo CA, Jr, Arias SA, et al. Suicide prevention in an emergency department population: The ed-safe study. JAMA Psychiatry 2017.
6. Richard O'Reilly MB, F.R.C.P.C. ,, Joan Bishop MD, F.R.C.P.C. ,, Karen Maddox RN, M.A. ,, Lois Hutchinson MD, F.R.C.P.C. ,, Michael Fisman MB, F.R.C.P.C. ,, Jatinder Takhar MD, F.R.C.P.C. ,. Is Telepsychiatry Equivalent to Face-to-Face Psychiatry? Results From a Randomized Controlled Equivalence Trial. Psychiatric Services 2007;58:836-43.
7. E. Anne Nelson, Mark E. Maruish, Joel L. Axler. Effects of Discharge Planning and Compliance With Outpatient Appointments on Readmission Rates. Psychiatric Services 2000;51:885-9.

APPENDICES

APPENDIX 1: Patient Navigation

This diagram shows the contact points among patients receiving care by the BH-VPN.

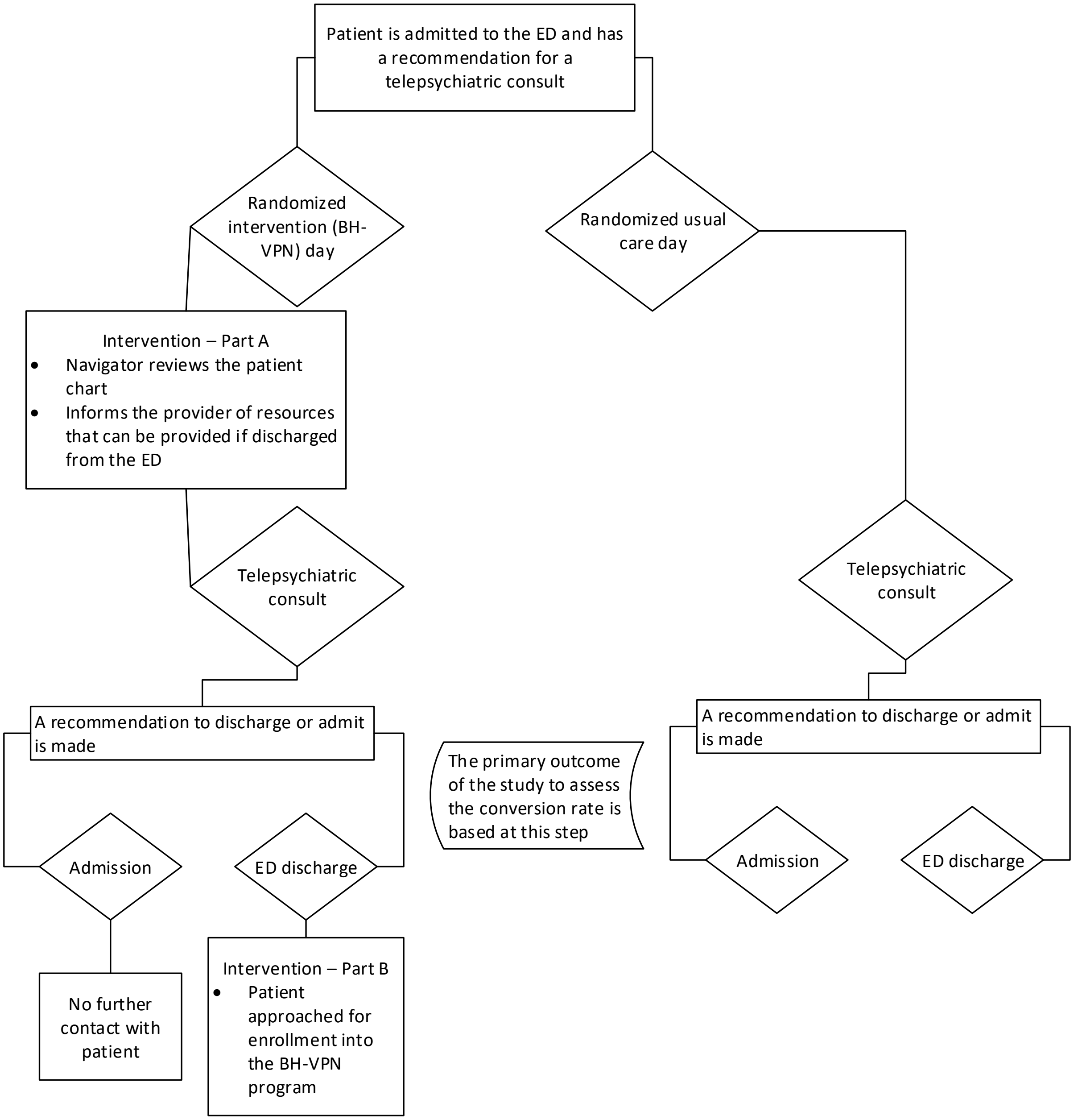

APPENDIX 2: Suicide Ideation steps

This diagram shows the procedure if a patient is identified with suicidal ideation from the CSSRS screening tool. This tool is administered during each phone contact.

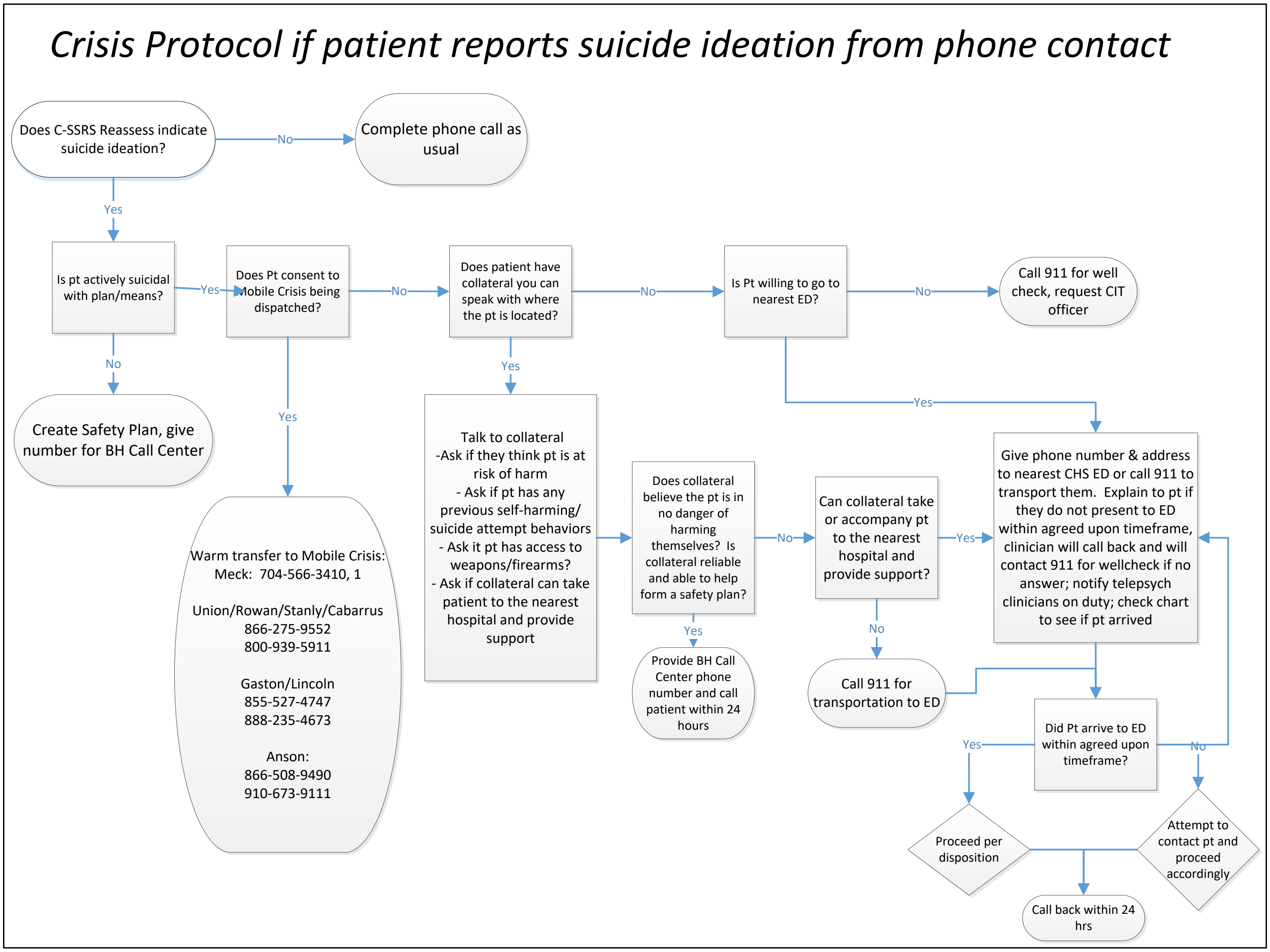

Supplement: Supplement 1. — Trial Protocol [file jamanetwopen-3-e1919954-s001.pdf]
