# Supplementary material for: Effect of a Virtual Patient Navigation Program on Behavioral Health Admissions in the Emergency Department: A Randomized Clinical Trial
Source: JAMA Netw Open. 2020 Jan 29;3(1):e1919954. doi: 10.1001/jamanetworkopen.2019.19954 (PMC6991284; doi:10.1001/jamanetworkopen.2019.19954)
Supplement: Supplement 2. — Data Sharing Statement [file jamanetwopen-3-e1919954-s002.pdf]

Data Sharing Statement

Roberge. Effect of a Virtual Patient Navigation Program on Behavioral Health Admissions in the Emergency Department. *JAMA Netw Open*. Published January 29, 2020. 10.1001/jamanetworkopen.2019.19954

Data
Data available: No
